# Coexpression of Fungal Cell Wall-Modifying Enzymes Reveals Their Additive Impact on Arabidopsis Resistance to the Fungal Pathogen, *Botrytis cinerea*

**DOI:** 10.3390/biology10101070

**Published:** 2021-10-19

**Authors:** Sivakumar Swaminathan, Nathan T. Reem, Vincenzo Lionetti, Olga A. Zabotina

**Affiliations:** 1 Roy J Carver Department of Biochemistry, Biophysics and Molecular Biology, Iowa State University, Ames, IA 50011, USA; sivaento@iastate.edu (S.S.); nathanreem@gmail.com (N.T.R.); 2 Dipartimento di Biologia e Biotecnologie “Charles Darwin”, Sapienza Università di Roma, 00185 Rome, Italy; vincenzo.lionetti@uniroma1.it

**Keywords:** cell wall, polysaccharides, *Arabidopsis thaliana*, *Aspergillus nidulans*, acetylesterase, feruloylesterase, *Botrytis cinerea*, biotic stress, defense-related pathways, additive effect

## Abstract

**Simple Summary:**

In the present study, we created transgenic Arabidopsis plants overexpressing two fungal acetylesterases and a fungal feruloylesterase that acts on cell wall polysaccharides and studied their possible complementary additive effects on host defense reactions against the fungal pathogen, *Botrytis cinerea*. Our results showed that the Arabidopsis plants overexpressing two acetylesterases together contributed significantly higher resistance to *B. cinerea* in comparison with single protein expression. Conversely, coexpression of either of the acetyl esterases together with feruloylesterase compensates the latter’s negative impact on plant resistance. The results also provided evidence that combinatorial coexpression of some cell wall polysaccharide-modifying enzymes might exert an additive effect on plant immune response by constitutively priming plant defense pathways even before pathogen invasion. These findings have potential uses in protecting valuable crops against pathogens.

**Abstract:**

The plant cell wall (CW) is an outer cell skeleton that plays an important role in plant growth and protection against both biotic and abiotic stresses. Signals and molecules produced during host–pathogen interactions have been proven to be involved in plant stress responses initiating signal pathways. Based on our previous research findings, the present study explored the possibility of additively or synergistically increasing plant stress resistance by stacking beneficial genes. In order to prove our hypothesis, we generated transgenic Arabidopsis plants constitutively overexpressing three different *Aspergillus nidulans* CW-modifying enzymes: a xylan acetylesterase, a rhamnogalacturonan acetylesterase and a feruloylesterase. The two acetylesterases were expressed either together or in combination with the feruloylesterase to study the effect of CW polysaccharide deacetylation and deferuloylation on Arabidopsis defense reactions against a fungal pathogen, *Botrytis cinerea*. The transgenic Arabidopsis plants expressing two acetylesterases together showed higher CW deacetylation and increased resistance to *B. cinerea* in comparison to wild-type (WT) Col-0 and plants expressing single acetylesterases. While the expression of feruloylesterase alone compromised plant resistance, coexpression of feruloylesterase together with either one of the two acetylesterases restored plant resistance to the pathogen. These CW modifications induced several defense-related genes in uninfected healthy plants, confirming their impact on plant resistance. These results demonstrated that coexpression of complementary CW-modifying enzymes in different combinations have an additive effect on plant stress response by constitutively priming the plant defense pathways. These findings might be useful for generating valuable crops with higher protections against biotic stresses.

## 1. Introduction

The plant cell wall (CW) is a dynamically active and highly controlled structure that is vital for plant growth and development [1]. The CW plays a crucial role in the determination of plant cell structure and shape of the tissues. Apart from its structural role, the CW is involved in important functions such as cell to cell interactions, growth and development of the whole plant, and interaction with the external environment [2]. The plant CW is mainly composed of cellulose and highly diverse heteropolysaccharides, such as hemicelluloses (xyloglucans, xylans and different mannans) and pectins (homogalacturonan, rhamnogalacturonan I, rhamnogalacturonan II, xylogalacturonan and apiogalacturonan), which are assembled in macromolecular networks [3,4]. In addition to the diverse monosaccharide composition, CW polysaccharides are also decorated with methyl, acetyl, and feruloyl groups, which are *O*-linked to sugars. These functional groups protect polysaccharides from the action of specific CW-degrading glycosyl hydrolases and also to cross link CW constituents for controlling cell extensibility [5,6,7].

Recently, it was demonstrated that plant CW alterations, either by intentionally impairing or overexpressing CW-related genes, have a significant effect on disease resistance and abiotic stresses [8,9,10,11]. It was initially assumed that the disease resistance phenotypes associated with alterations of CW integrity were due to the incapability of un-adapted pathogens to overcome the modified CW compositions/structures in the genetically modified plant mutants or over-expressed transgenic lines. However, later studies found that the CW is not just a passive barrier. CW alterations trigger complex defensive signaling pathways to fight against plant pathogens [11,12,13,14,15]. The role of CW polysaccharides on plant resistance to pathogens has been extensively reviewed recently in detail [16].

Based on research evidence, it was assumed that artificial CW modifications might induce plant defense responses even prior to pathogen infection, and these defense reactions could be able to reduce pathogen infection and spread [16]. Several efforts have been made to unravel the complexity behind the role of the CW in plant pathogen resistance [7,17]. Indeed, increasing evidence shows that changes in CW composition, via either altering polysaccharide biosynthesis or polysaccharide post-synthetic modifications in muro, induced reactions similar to those induced during plant responses to naturally occurring biotic/abiotic stresses [7,16,17,18,19,20,21,22,23].

Substantial experimental evidences have proven that modifications in pectin biosynthesis strongly affected the plant response to pathogens. For instance, it was found that the nuclear-localized transcriptional activator AtERF014 can act as a dual regulator of *Arabidopsis* resistance against two pathogens, *Pseudomonas syringae* and *B. cinerea* [24]. Altering the expression of AtERF014, either by overexpressing or by gene-silencing, influenced the expression of pectin biosynthesis genes and also the pectin content, which resulted in differential resistance or susceptible response of Arabidopsis plants against these two pathogens [24]. Arabidopsis *gae1 gae6* double mutants, which have terminated expression of two glucuronate 4-epimerases involved in the synthesis of the pectin precursor UDP-D-galacturonic acid, showed susceptibility to the pathogens *P. syringae* and *B. cinerea* [25]. The Arabidopsis double mutant *powdery mildew-resistant* 5 and 6-3 (*pmr5pmr6-3*), which showed a pectin-enriched CWs phenotype, was found more susceptible to *P. syringae,* and interestingly was found more resistant to the fungus *Colletotrichum higginsianum* than WT plants [26,27,28]. Increased susceptibility to *C. higginsianum* was also observed in Arabidopsis *mur8-1* mutants, which displayed a reduction in CW rhamnose and also RG-I content, in comparison to the WT [26].

Pectic complexity is further amplified by post-synthetic modifications such as acetylation, methylesterification, etc. [29]. Specific endogenous pectin methylesterases (PME) and PME inhibitors (PMEI) were found dynamically modulated during plant–microbe interactions [30,31]. Necrotrophic pathogenetic microbes have evolved the capability to degrade lowly methylesterified pectin efficiently by using specific polygalacturonases (PGs). At later stages of pathogen infection, plants can defend their CW integrity by inducing specific PMEIs to block the PME-mediated demethylesterification of pectin. This action protects the CW from further degradation by PGs and limits pathogen spread [21]. Accordingly, Arabidopsis, wheat and cotton plants overexpressing pectin methylesterases inhibitors (PMEI) exhibited increased resistance to *B. cinerea*, *Fusarium graminearum* and *Verticillium dahliae*, respectively [32,33,34]. The overexpression of PG-inhibiting proteins (PGIPs) in different plant species increased their resistance to necrotrophic fungi and bacteria [35,36]. Additionally, it was demonstrated that reduced pectin acetylation in Arabidopsis by overexpression of *Aspergillus nidulans* acetylesterase (AnRAE) resulted in the induction of specific defensive responses and increased resistance to *B. cinerea* [37].

The molecular biology of pectin is highly complex, and we are still far away from understanding the exact details of their contribution to CW integrity-mediated pathogen resistance. However, most of the resistance phenotypes involving altered pectic composition and structures were partially found to be associated with enhanced accumulation of oligogalacturonides (OGs), which are known to be damage-associated molecular patterns (DAMPs) resulting from the breakdown of pectic α-galacturonosyl residues. OGs have been recognized in Arabidopsis by Wall-Associated Kinase 1 (WAK1), which serves as a pattern recognition receptor in triggering plant immune responses [38]. OGs were demonstrated to elicit a wide array of defense responses, including a strong apoplastic oxidative burst, accumulation of phytoalexins and up-regulation of pathogen defense-related genes which subsequently confers resistance to pathogens [39,40,41,42,43].

Some experimental evidence demonstrated the existence of a link between the alteration of xylose content in hemicellulose and their degree of acetyl esterification to the immune response of Arabidopsis to plant pathogens. For instance, plants with higher levels of wall-bound xylose, as occurs in the Arabidopsis *de-etiolated3* (*det3*) and *irx6* mutants [44,45] and the *xyl1-2* mutant [46], had enhanced resistance to the fungus, *P. cucumerina*. On the other hand, Arabidopsis, *agb1* and *agg1 agg2* mutants, which have reduced xylose content were found to be highly susceptible to *P. cucumerina* [47,48,49]. The Arabidopsis *Reduced Wall Acetylation2* (*rwa2*) mutant, with 20% reduced polysaccharide O-acetylation, was found more resistant to the necrotroph, *B. cinerea* and the biotroph, *Hyaloperonospora arabidopsidis* than WT plants [50,51]. Similarly, transgenic plants over-expressing a fungal xylan acetylesterase showed a reduction in CW xylan acetylation, and consequently these plants were found highly resistant to necrotrophic fungi [37]. The Arabidopsis *pmr5* mutant, with impaired O-acetylation of wall polysaccharides, was found more resistant to the pathogens, *Erysiphe cichoracearum* and *C. higginsianum* than WT plants [26,27,52].

Arabidopsis *esk1* plants with impaired xylan acetylation showed variations in their cellular biochemical compositions, such as increased accumulation of abscisic acid, constitutive upregulation of the genes encoding antimicrobial peptides and the enzymes involved in the synthesis of tryptophan-derived metabolites, enhanced accumulation of disease resistance-related secondary metabolites and different osmolytes, which overall resulted in plant resistance to freezing, drought, and salinity [53,54,55,56].

It was also shown that ferulic acid (FA) plays an important role in plant–pathogen interactions, and that many phenolic compounds such as FA are often induced in response to various biotic stresses. FA is known to play a vital role in fungal pathogen resistance and has also been found to act as an insect deterrent [57,58]. Experiments showed that FA-mediated cross-linking results in CW stiffening and a reduction in growth [59], and that a negative correlation exists between the amount of CW feruloyl esterification and pathogen infection [60]. In another experiment, Arabidopsis and Brachypodium (*Brachypodium distachyon*) plants expressing *A*. *nidulans* feruloylesterase, which hydrolyzes ester linkages between host plant CW polysaccharides and FA, were found to have reduced CW feruloylation and increased susceptibility to the pathogens *B. cinerea* and *Bipolaris sorokiniana*, respectively [61].

In our previous studies, in Arabidopsis and Brachypodium plants we investigated the consequences of the expression of two different fungal acetylesterases, *A. nidulans* xylan acetylesterase (AnAXE) and a rhamnogalacturonan acetylesterase (AnRAE), and both the enzymes remove acetyl groups (deacetylates) from CW polysaccharides [37]. The transgenic plants with reduced polysaccharide acetylation showed significantly increased resistance to the fungal pathogens *B. cinerea* and *B. sorokiniana*, respectively. In another related study in Arabidopsis and Brachypodium plants, we investigated the effect of expression of an *A. nidulans* feruloylesterase (AnFAE), which removes feruloyl groups (deferuloylates) from CW polysaccharides [61,62]. Transgenic Arabidopsis and Brachypodium plants expressing AnFAE showed significantly reduced levels of FA and wall-associated extensins, and increased susceptibility to *B. cinerea* and *B. sorokiniana*, respectively [61,62].

Earlier studies showed that naturally occurring acetylation and feruloylation of CW polysaccharides plays an important role in protecting the CW from the action of specific CW-degrading glycosyl hydrolases and also strengthening CW via diferulic cross-linkages [5,6,7]. It was shown that CW deacetylation increased plant resistance [37,50,51,52,53,54,55,56,62] by increasing accessibility of CW polysaccharides to plant glycosidases for partial degradation. The oligosaccharides released as a result of this partial degradation can be perceived as DAMPs and trigger defense-related genes. Furthermore, previous studies have shown that expressing fungal acetylesterases resulted in the induction of defense pathway genes even without pathogen inoculation [37,61]. On the other hand, expression of feruloylesterase resulted in significantly reduced levels of FA and wall-associated extensins, thereby weakening the CW. Deferuloylation of the CW did not have an impact on the expression of defense related genes in non-infected transgenic plants but increased their susceptibility to *B. cinerea* and *B. sorokiniana* [61,62], most likely due to mechanical weakness of deferuloylated CWs.

Overall, the results obtained earlier showed that both deacetylation by acetylesterases and deferuloylation by feruloylesterase produced opposite effects on plant resistance to pathogens [37,61] due to different consequences of their actions. The expression of fungal acetylesterases caused constitutive priming of plant defense pathways even before pathogen infection and made the plants be in a ready state to defend against the possible microbial attacks.

In the present study, we generated double transgenic plants over-expressing the same fungal acetylesterases (AnAXE and AnRAE) [37], either together or in combination with the previously used feruloylesterase (AnFAE) [61], to investigate the impact of stacking two beneficial genes (*AnAXE/AnRAE*) to improve plant immunity and also to investigate the effect of two genes that were known to produce opposite effects (*AnAXE/AnFAE* and *AnRAE/AnFAE*). We demonstrated that over-expressing stacked genes (*AnAXE/AnRAE*) produced an additive impact on induction of Arabidopsis defense signaling pathways and additive impact on defense reactions against *B. cinerea*. The positive effect of reduced CW acetylation by AnAXE or AnRAE on plant resistance was also able to compensate the negative impact of weakened CW due to reduced feruloylation caused by AnFAE. These results provide new insights into the application of post-synthetic modification of the CW as a tool to protect valuable crops against pathogens.

## 2. Materials and Methods

### 2.1. Arabidopsis Growth Conditions

Arabidopsis seeds of different genotypes were planted and grown on LC-1 potting soil mix (Sun Gro Horticulture, Agawam, MA, USA) in a growth chamber under controlled conditions. The growth chamber was maintained at 21 °C, with 16 h light/8 h dark photoperiod, 65% relative humidity and 160 µmols^−1^ m^−2^ light intensity [37,61].

### 2.2. Generation of Transgenic Plants

In this study, the UBIQUITIN-10 promoter (UBQ10), which is an endogenous, constitutively expressed promoter of Arabidopsis, was used instead of a commonly used CaMV 35 S promoter [63]. Recently, a set of Gateway-compatible binary vectors were developed for the expression of transgenes tagged with different fluorescent proteins [64]. Of these binary vectors, pUBC-CFP, which enables the tagging of proteins with cyan fluorescent protein (CFP) at the C-terminus, was chosen for the present investigation [64]. pUBC-CFP possesses a gene conferring resistance to the Basta herbicide for selection of homozygous Arabidopsis transformants. The DNA sequences of *AnAXE, AnRAE,* and *AnFAE* genes were first cloned into the pENTR/D-TOPO entry vector and later Gateway-recombined into the pUBC-CFP vector. The Arabidopsis β-expansin signal peptide was fused to the N-terminus of each gene [37,61,62] to ensure the protein secretion into the apoplastic space to aid in the deacetylation and deferuloylation of CW polysaccharides. The genes containing the signal peptide sequences were inserted in between the promotor and CFP sequences. To generate single mutants, the recombinant pUBC-CFP vectors carrying *AnAXE, AnRAE*, and *AnFAE* were transformed separately into *Agrobacterium tumefaciens* cells (EHA101 strain) by electroporation and transformed onto WT Arabidopsis plants (Col-0) by the floral-dip method [65].

To create three combinations of transgenic plants over expressing two genes together, two selection methods were required, one for each transgene. So far, neither of the vectors generated by Grefen et al. [64], nor any other available vector source, possessed a Gateway-compatible UBQ10 promoter with selection other than resistance to Basta. Thus, a vector with different selection marker was created. To achieve this, Gibson assembly [66] protocol was used to transfer the currently available expression cassette from pUBC-CFP vector onto an existing backbone: pCAMBIA-1300-MCS (Figure S1). Complementary overhanging primers for each cassette inserts and plasmid backbones were designed, and fragments were amplified by PCR, annealed together, and later transformed into *E. coli*. Then, basta-resistant transgenic plants already containing single genes, pUBC-[*AnAXE/AnRAE/AnFAE*]-CFP, were transformed with Agrobacterium carrying pCAMBIA-UBQ10-[*AnAXE/AnRAE/AnFAE*]-CFP by the floral-dip method. After transformation, three independent transgenic Arabidopsis lines were selected for each construct, and homozygous plants were generated using herbicide and/or antibiotic resistance. PCR was carried out with genomic DNA to confirm the presence of full-length constructs.

### 2.3. RNA Extraction, cDNA Synthesis, and Real-Time qPCR

From the leaves of 3-week-old plants, total RNA was extracted using the SV Total RNA Isolation System (Promega Corp, Madison, WI, USA), and cDNA was synthesized with the SuperScript III First Strand Synthesis System (Invitrogen Corp, Carlsbad, CA, USA). Real-time quantitative PCR (RT-qPCR) was carried out on the Mx3000P qPCR System (Agilent, Santa Clara, CA, USA), using the GoTaq qPCR Master Mix (Promega Corp, Madison, WI, USA), and appropriate primers (Table S1) to estimate the relative expression of various genes. The *ACTIN2* gene (*At3g18780*) was used as the reference gene. For a particular gene, the expression level in WT plant was set as 1, and the expression of that gene in transgenic plants was calculated relative to that of WT plants. The comparative threshold cycle technique [67] was used for estimating differences between the transcript level in WT and transgenic plants.

The transcript levels of various defense genes were estimated using RT-qPCR in healthy uninfected Arabidopsis plants as described earlier [37,61]. The expression levels of defense pathway genes tested in our study were: jasmonic acid responsive gene1 (*JR1*), phytoalexin deficient3 (*PAD3*) gene, β-1,3-endoglucanase2 (*bG2*) gene, pathogenesis-related proteins 1 and 5 (*PR1* and *PR5*) genes, plant defensin1.2 (*PDF1.2*) gene, wound responsive3 (*WR3*) gene, pathogen-induced W box-containing transcription factor (*WRKY40*) gene, polygalacturonase-inhibiting protein1 (*PGIP*) gene, *CYP81F2* (*CYP*) gene, and oxidoreductase (*RetOx*) gene [37,61].

### 2.4. CW Composition Analysis

CWs of different Arabidopsis genotypes were extracted from healthy uninfected plants as described earlier [37]. Acetyl content in the CW was estimated using an assay previously developed by McComb and McCready (1957) [68]. Briefly, 10 mg of CW was weighed and mixed with 2.5 mL of 0.5 M hydroxylamine hydrochloride by vortexing. Subsequently, 2.5 mL of 2 N sodium hydroxide was added gradually and mixed thoroughly. From the above mixture, 0.5 mL was aliquoted and mixed with 0.2 mL of water and further with 0.5 mL of acid methanol (35.2 mL 70% perchloric acid in 500 mL of methanol). Next, 1.3 mL of ferric perchlorate was added in small increments and mixed after each addition. The mixture was incubated at room temperature for 15 min and the resultant ‘ferric acetohydroxamic complex’ product was estimated by spectrometric measurement of absorbance at 510 nm. Simultaneously, a standard curve was generated using Glc pentaacetate as a standard. Then, the acetyl content of the samples was calculated using the standard curve.

For the determination of ferulic acid content, total phenolic acids were first extracted from the CWs of various samples and analyzed further using the method as described earlier [62]. Briefly, 1 mg of CW was weighed and incubated in 2 mL of 2M NaOH for 24 h. The supernatant was collected, the reaction was repeated one more time, and the resultant supernatants were combined together. The supernatant was neutralized with HCl and the total phenolics were extracted using ethyl acetate, which was then evaporated to dryness with a stream of N_2_. The phenolics were redissolved in 100% methanol and analyzed on reverse-phase HPLC using a Prevail C18 5 µ column (4.6 mm × 250 mm; Grace Davison Discovery Sciences, Deerfield, IL, USA) under UV detection at 290 nm and 320 nm. The phenolic acids were separated using a gradient of 0.1% trifluoroacetic acid in water (pH 2.8) and acetonitrile at a flow rate of 1 mL min^−1^ under following conditions: 0–10 min—95% water; 10–30 min—85% water; 30–40 min—70% water; 40–47 min—5% water; 47–55 min—95% water. To determine the amount of ferulic acid content, a standard curve was generated using different concentrations of standard ferulic acid (Sigma-Aldrich, St. Louis, MO, USA).

### 2.5. Infection of Arabidopsis Genotypes with Botrytis Cinerea

*Botrytis cinerea* (strain SF1) was grown for 15 days on potato dextrose agar (39 g L^−1^) plates at 23 °C with a 12 h light and 12 h dark photoperiod, as mentioned previously [32]. The conidial spores produced were harvested by gently washing the surface of the agar plates with 5 mL of sterile water. The spore suspension was filtered through glass wool to remove the residual mycelia, and the spore concentration was estimated using a Thoma chamber. To achieve uniform spore germination, the conidia were grown in potato dextrose broth (24 g L^−1^) at room temperature for 3 h and the germinated spores were used for inoculation of Arabidopsis leaves, as described previously [50,69]. Fully developed uniform leaves were cut from 3-week-old Arabidopsis plants (three leaves per plant). The petioles of cut leaves were embedded within 0.8% agar in square Petri dishes. Two 5 µL droplets of germinated spore suspension (5 × 10^5^ conidia mL^−1^) were placed on each side of the mid-rib on the surface of each leaf. Potato dextrose broth without spores was used as negative control. The inoculated leaves were incubated at 24 °C with a 12 h light and 12 h dark photoperiod, and the lesion diameter was measured using ImageJ software [70] 48 h post inoculation (hpi).

### 2.6. Determination of H_2_O_2_ Accumulation

Healthy uninfected WT and transgenic leaves were assayed for H_2_O_2_ production using the 3,3′-diaminobenzidine (DAB) staining method as described previously [71]. DAB solution was prepared first (1 mg/mL; pH 3.0), and subsequently Tween-20 and Na_2_HPO_4_ were added to a final concentration of 0.05% and 10 mM, respectively. Three uniform rosette leaves from 3-week-old Arabidopsis plants were harvested and immersed in 5 mL of DAB solution mixture in a 24-well plate. Then, the leaf samples were vacuum infiltrated for 5 min twice to ensure DAB solution penetrated the tissues. After vacuum infiltration, the 24-well plate was covered with aluminum foil and placed on a shaker for 12 h. After 12 h, the leaves were transferred to a bleaching solution (3:1:1 ethanol: acetic acid: glycerol) and boiled at 95 °C for 15 min. After boiling, the old bleaching solution was replaced with the fresh bleaching solution and the DAB-stained leaves were photographed.

### 2.7. Statistical Analysis

All experimental mean data were subjected to statistical analysis using R software. The bioassay data of total acetyl content, ferulic acid content and lesion size data of each experiment were initially tested for significance by analysis of variance (ANOVA). Subsequently Fisher’s protected least significant difference (LSD) test value was used to compare treatment means at *p* < 0.05. The statistical significance of the relative transcript of RT-qPCR data of different treatments was established using Student’s *t*-test at *p* < 0.05 (*) and also at *p* < 0.01 (**).

## 3. Results

### 3.1. Generation of Transgenic Arabidopsis Plants Expressing A. nidulans Acetylesterases (AnAXE and AnRAE) and Feruloylesterase (AnFAE)

In this study, six different transgenic lines were generated as follows: transgenic Arabidopsis lines expressing three single genes separately (*AnAXE, AnRAE* and *AnFAE*) and three transgenic Arabidopsis lines each co-expressing two genes together (*AnAXE/AnRAE, AnAXE/AnFAE* and *AnRAE/AnFAE*). Transgenic Arabidopsis lines expressing single genes were generated by employing pUBC-CFP binary vector with a C-terminal CFP fusion under the control of endogenous UBIQUITIN-10 promoter (UBQ-10) (Figure S1). Native UBQ-10 was chosen over the 35 S promoter since it facilitates moderate expression in nearly all Arabidopsis tissues. Moreover, using UBQ-10 avoids possible problems of gene silencing associated with 35 S promoter [63].

In order to confirm the gene expression in all transgenic lines, RT-qPCR analysis was conducted (Figure S2). All the selected lines showed that the introduced genes’ transcript expression was at a comparable level without statistically significant differences. All transgenic plants showed no differences in growth and development with respect to control plants.

### 3.2. Expression of Fungal Acetylesterases Reduced the Degree of CW Acetylation in Transgenic Arabidopsis

**Figure 1:**
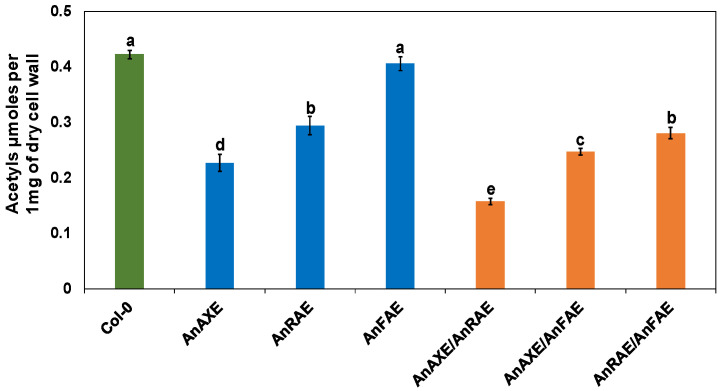
Cell wall acetylation of transgenic and WT plants (Col-0). Acetyl content is represented in µmol per mg dry CW material. Data represent the average ± SD of three different independent transgenic plants of each construct and three WT plants. Letters (a–e) indicate the significant differences among the genotypes (Fisher’s LSD test, *p* < 0.05).

The effect of expression of acetylesterases on the degree of acetyl esterification of CW polysaccharides was evaluated. The acetyl groups were extracted from the CWs of leaves of the transgenic and WT plants and total acetyl contents were quantified by spectrophotometric assay [68]. Our results indicated that AnAXE and AnRAE plants showed a 45% and 33% reduction in CW acetyl levels, respectively, in comparison to Arabidopsis WT plants (Col-0) (Figure 1). As expected, the acetyl content in CWs of AnFAE plants was equal to Col-0 plants. Among the double-gene overexpressor transgenic Arabidopsis plants, AnAXE/AnRAE showed the greatest reduction in acetyl content (66%), followed by AnAXE/AnFAE with a 45% reduction, and AnRAE/AnFAE with a 39% reduction in acetyl contents (Figure 1).

### 3.3. Transgenic Arabidopsis Expressing Fungal Feruloylesterase Showed Reduction in CW Feruloylation

**Figure 2:**
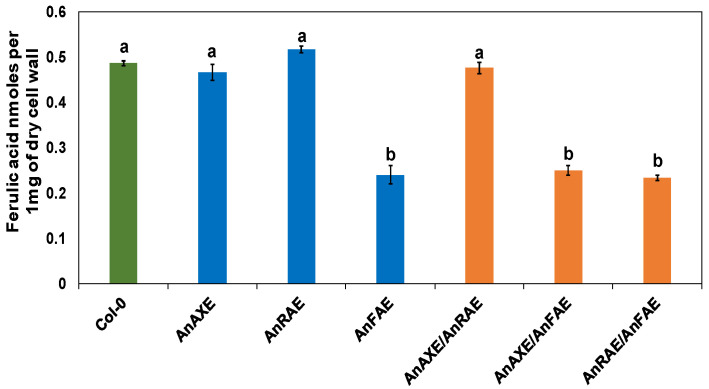
Cell wall feruloylation of transgenic and WT (Col-0) Arabidopsis plants. Ferulic acid content is represented in nmol per mg dry CW material. Data represent the average ± SD of three different independent transgenic lines of each construct and three WT plants. Letters (a and b) indicate significant differences among the genotypes (Fisher’s LSD test, *p* < 0.05).

The levels of ferulic acid content were quantified in transgenic plants expressing feruloylesterase in comparison to WT plants. The degree of feruloylation was determined in CWs extracted from transgenic and WT plants using reverse-phase HPLC and commercial ferulic acid as a standard. The transgenic plants AnFAE, AnAXE/AnFAE and AnRAE/AnFAE showed a 51%, 49%, and 53% reduction in ferulic acid content, respectively, in comparison to Col-0 plants (Figure 2). However, the AnAXE, AnRAE and AnAXE/AnRAE plants showed the same level of ferulic acid content as that of Col-0 plants.

### 3.4. Acetylesterase Expressing Plants Have Increased Resistance to Necrotrophic Fungal Pathogen

Our previous studies with single gene-expressing Arabidopsis plants showed that deacetylation increased resistance against the fungal pathogen *B. cinerea* whereas deferuloylation increased the susceptibility [37,61]. The effect of ectopically expressing either single genes (*AnAXE, AnRAE*, and *AnFAE*), or pairs of genes (*AnAXE/AnRAE*, *AnAXE/AnFAE*, and *AnRAE/AnFAE*) in Arabidopsis plants was assessed by inoculating leaves with *B. cinerea* conidia and observing lesion symptom development 48 hpi with ImageJ software.

**Figure 3:**
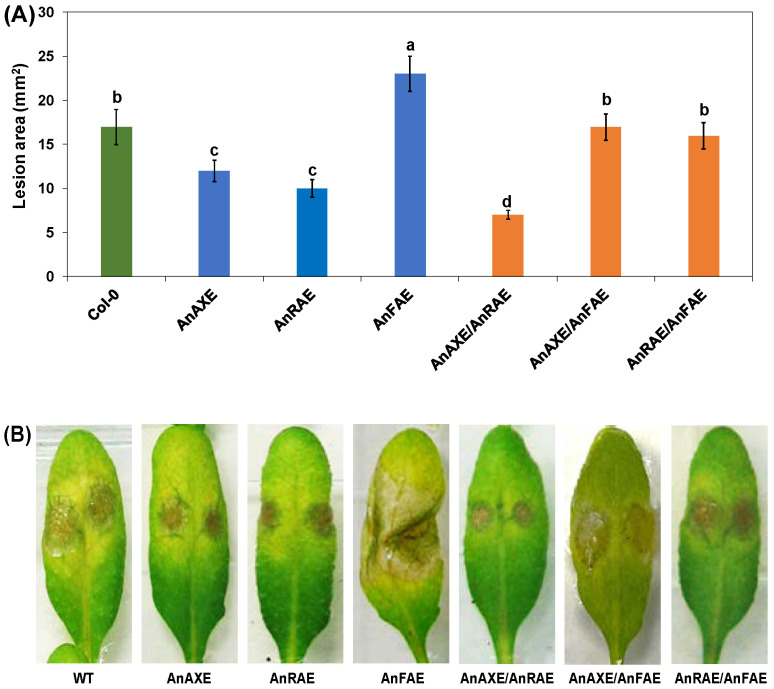
Bioassay of Arabidopsis transgenic plants against the fungal pathogen, *Botrytis cinerea*. Three-week-old uniform Arabidopsis leaves were inoculated with 5 µL droplets of fungal spores (5 × 10^5^ conidia mL^−1^) and examined 48 h post inoculation (hpi). (**A**) Measurement of lesion size on Arabidopsis leaves (48 hpi). Lesion sizes were measured by ImageJ software. Letters (a--d) indicate the significant differences among the genotypes (Fisher’s LSD test, (*p* < 0.05). (**B**) Extent of symptom development due to *B. cinerea* infection on Arabidopsis leaves (48 hpi; representative images shown).

Among the single-gene overexpressing transgenic Arabidopsis plants, AnAXE and AnRAE plants showed resistance to the spread of fungal pathogens with a 29% and 32% reduction in lesion size, respectively, in comparison to Col-0 (Figure 3A). AnFAE Arabidopsis plants were highly susceptible to *B. cinerea,* and the lesion size was 14% larger than the WT Arabidopsis. AnAXE/AnRAE Arabidopsis plants showed the highest resistance to the pathogen among all the plants with a 60% reduction in lesion size. AnAXE/AnFAE and AnRAE/AnFAE Arabidopsis plants were found to be susceptible to the pathogen at the same level as that of the WT Arabidopsis (Figure 3A,B).

### 3.5. Deacetylation and Deferuloylation of CW Resulted in Expression of Pathogen-Responsive Genes

**Figure 4:**
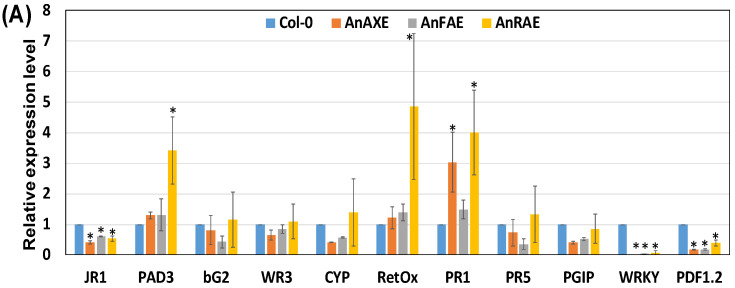

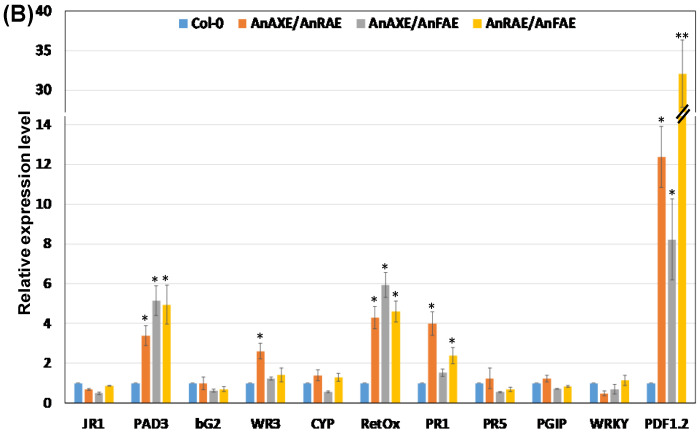
Overexpression of acetyesterases and feruloylesterase trigger pathogen-responsive gene expression. RT-qPCR analysis was conducted to find out the transcript level of defense pathway genes in six different uninfected healthy transgenic lines: (**A**) Transcript level of single gene overexpressor plants (AnAXE, AnRAE, and AnFAE); (**B**) Transcript level of double gene overexpressor plants (AnAXE/AnRAE, AnAXE/AnFAE and AnRAE/AnFAE). Three-week-old uninfected healthy seedlings were subjected to transcript analysis. *ACTIN2* was used as reference gene to normalize the data [37,61]. The transcript data represents average ± SD of three different independent transgenic lines for each construct and WT plant. Asterisks indicate significant differences among the transgenic plants and WT plants (Student’s *t* test, * *p* < 0.05; ** *p* < 0.01).

To determine whether overexpression of acetylesterases/feruloylesterase and the resultant decrease in CW acetylation/feruloylation constitutively primed the plant defense pathways, transcript levels of defense pathway-related genes were measured by RT-qPCR in healthy, uninfected Arabidopsis plants. In Arabidopsis transgenic plants expressing *AnRAE* gene, the upregulation of *PAD3*, *RetOx*, and *PR1* transcripts was found to be 3.5-fold, 5-fold, and 4-fold higher than WT plants, respectively (Figure 4A). There was a 3-fold induction of *PR1* gene transcript level in AnAXE-expressing plants. There were no genes induced in AnFAE plants. At the same time, the transcript level of *WRKY* and *PDF1.2* genes were significantly downregulated by 6- to 10-fold in all three single-gene Arabidopsis transgenic plants (Figure 4A).

Among the three double-gene-expressing Arabidopsis transgenic plants, the transcript level of *PAD3*, *RetOx*, *PR1* and *PDF1.2* genes were upregulated significantly in comparison to the WT plants (Figure 4B). Interestingly, *PDF1.2* transcript level was upregulated to a higher level than any other gene by 12.5-fold, 8-fold, and 32.5-fold in AnAXE/AnRAE, AnAXE/AnFAE and AnRAE/AnFAE plants, respectively. The transcript level of *PAD3*, *RetOx* and *PR1* genes were upregulated by 2- to 6-fold on an average in the three transgenic plants. *CYP* showed 2.5-fold higher expression in AnAXE/AnRAE plants (Figure 4B).

### 3.6. Acetylesterase Expressing Plants Have Enhanced H_2_O_2_ Accumulation

**Figure 5:**
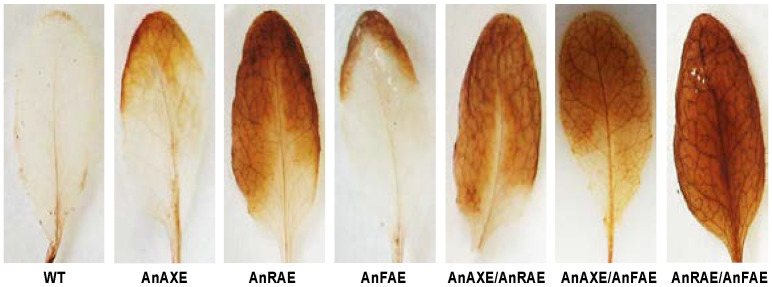
Detection of H_2_O_2_ accumulation in uninfected healthy transgenic plants. DAB staining assay was carried out on uninfected three-week-old WT and transgenic leaves (representative images shown).

To further investigate the correlation between the induction of the *RetOx* transcript noticed in transgenic lines (Figure 4) and the resultant accumulation of H_2_O_2_ in the uninfected healthy leaves, DAB staining assay was carried out. Increased accumulation of H_2_O_2_ was observed in AnRAE, AnAXE/AnRAE, AnAXE/AnFAE and AnRAE/AnFAE plants in comparison with WT plants, which positively correlates with RetOX transcript level data (Figure 4 and Figure 5).

## 4. Discussion

Plants are under continuous biotic and abiotic stresses that can severely compromise their survival, and the plant CW and its polysaccharides acts as mechanical barriers to give protection against stresses. Compromising plant CW integrity, by either altering polysaccharide biosynthesis or polysaccharide post-synthetic modifications, results in high impact on disease resistance and abiotic stresses [16,17,18,19,20,21,22,23,37]. Such deliberately introduced CW modifications could induce plant defense pathway gene(s) and defense responses even prior to pathogen infection, and these defense responses might be able to reduce or slow down pathogen spread during actual infection [16].

In previous studies, we demonstrated that the expression of two different fungal acetylesterases (AnAXE and AnRAE) individually, resulted in reduced polysaccharide acetylation and increased plant resistance to two pathogens, *B. cinerea*, and *B. sorokiniana* [37]. On the other hand, transgenic over expression of the fungal feruloylesterase (AnFAE) showed a substantial reduction in CW ferulic acid and increased susceptibility to *B. cinerea* and *B. sorokiniana* [61,62]. Deacetylation resulted in exposure of the CW polysaccharides to CW-degrading hydrolases, possibly leading to the generation of DAMPs and constitutive priming of plant defense pathways, which might have prepared the plants in a ready state to defend against the possible attack of pathogens in the future [37].

In this present study, we generated transgenic plants over expressing previously reported *AnAXE, AnRAE,* and *AnFAE* genes in pairwise combinations in order to mainly analyze the effect of stacking genes on CW modifications, Arabidopsis defense reactions against *B. cinerea,* and the induction of defense pathway-related genes. The obtained results from this study demonstrated the additive impact of stacked *AnAXE/AnRAE* genes in enhancing the Arabidopsis defense response against the fungal pathogen *B. cinerea*.

In our previous studies, we overexpressed AnAXE, AnRAE, and AnFAE under the 35 S promoter and did not observe any abnormality in the phenotype or physiology of the plants in over expressing AnAXE, AnRAE, or AnFAE under the 35 S promoter [61,62]. In this work, an endogenous UBQ10 promoter was chosen, instead of the 35 S promoter, for both single and double overexpressors in order to promote a moderate constitutive co-expression of genes in nearly all Arabidopsis tissues [63]. This strategy also avoids possible problems of co-suppression or gene silencing associated with the 35 S promoter, which have been previously reported [63]. Likewise, in this study, no abnormality in phenotype or physiology were detected in either single or double overexpressors. Further, the RT-qPCR results showed that the expression level of the overexpressed genes in different transgenic plants were at a comparable level with no significant difference observed under the control of UBQ10 promoter (Figure S2).

The results showed that AnAXE and AnRAE plants had reduced acetyl contents (Figure 1) and AnFAE plants had reduced ferulic acid contents in the CW (Figure 2). AnAXE and AnRAE-expressing plants showed higher resistance to *B. cinerea*, whereas AnFAE-expressing plants were more susceptible to the fungus. All these data obtained were in accordance with our previous results obtained in Arabidopsis plants expressing the same genes under the control of the 35 S promoter [37,61,62]. This shows that the native UBQ10 promoter provides a sufficient level of the introduced gene expression, which supports a high level of enzymatic activity localized in the apoplast, comparable to 35 S-driven expression.

AnFAE transgenic plants were found to be highly susceptible to the fungal pathogen *B. cinerea* (Figure 3). Our previous study showed that deferuloylation by AnFAE reduced the strength of the CW cross-linking, which resulted in susceptibility of transgenic plants to *B. cinerea* [61]. The susceptibility of AnAXE/AnFAE and AnRAE/AnFAE plants was not significantly different from WT plants (Figure 3), indicating that the expression of AnAXE or AnRAE can revert the increased susceptibility caused by AnFAE expression.

Pyramiding beneficial compatible genes and quantitative trait loci (QTL) through natural breeding or transgenic methods was beneficial for the development of superior resistance against plant pathogens [72,73,74,75]. In our study, the double-transgenic AnAXE/AnRAE plants were found to be highly resistant to *B. cinerea* with respect to the single AnAXE or AnRAE overexpressors. The level of resistance positively correlates with the level of reduction in CW acetyl content (Figure 1 and Figure 3). This shows a complementary additive effect of both *AnAXE* and *AnRAE* genes when expressed together. Interestingly, the combination of acetylesterase genes with feruloylesterase reverses the negative impact of CW deferuloylation on plant resistance.

Earlier studies showed that CW deacetylation primed plant resistance [37,50,51,52,53,54,55,56,62]. It was proposed that deacetylated CW polysaccharides become more accessible to plant glycosidases for partial degradation, thus possibly producing DAMP oligosaccharides. The DAMPs triggered defense-related genes [37]. On the other hand, the deferuloylation of the CW resulted in decreased plant defense resistance due to mechanically weakened CW [61]. The reduction in CW cross-linking via FA and the reduction in wall-associated extensins by deferuloylation resulted in a mechanically weakened CW [61]. Contrary to deacetylation, the deferuloylation of the CW did not trigger defense-related genes [61]. Both the earlier studies and the present study showed that deacetylation by acetylesterases and deferuloylation by feruloylesterase are independent processes and produced opposite effects on plant resistance to pathogens due to different consequences of their actions [37,50,51,52,53,54,55,56,61,62]. The reduction in polysaccharide feruloylation weakens the CW, which results in a more digestible biomass, which is the goal of the contemporary bioenergy industry, but at the same time makes it easier for fungal penetration and invasion into plants [61,76,77]. However, the addition of one acetylesterase and, thus, the induction of defense-related genes can compensate for the CW weakness and assist in plant protection against pathogens.

Our results suggest the possibility to enhance plant immunity through a coordinated expression of genes that produce different CW modifications. An earlier example of successful application of this approach comes from experiments in wheat. The combination of PvPGIP2 and TAXI-III, a xylanase inhibitor, enhanced wheat host resistance against *F. graminearum* [78]. Additionally, pyramiding two pectinase inhibitors, PvPGIP2 and PMEI, resulted in broad spectrum pathogen resistance [78].

The CW deacetylation of transgenic plants might result in high exposure of polysaccharides to endogenous CW-degrading enzymes, such as polygalacturonases (PGs) and hemicellulases. Our earlier studies showed that the deacetylated CWs of AnAXE and AnRAE transgenic plants were highly susceptible to a xylanase and PG, respectively [37,61,62]. The activity of these enzymes might have resulted in the constitutive generation of OGs or other DAMPs, triggering defense-related pathways and priming transgenic plants to defend against *B. cinerea*. Several studies indicated that CW-mediated plant resistance was associated with an enhanced accumulation of DAMP oligomers [16,18,19,22,79]. Changes in expression of different defense pathway genes in both single and double-gene overexpressor transgenic plants support these observations (Figure 4). Transcript levels of *PAD3, RetOx, PDF1*.2 and *PR1* were induced in all three double-gene transgenic plants (AnAXE/AnRAE, AnAXE/AnFAE and AnRAE/AnFAE) and *WR3* gene was induced in AnAXE/AnRAE plants (Figure 4B). In single-gene transgenic AnRAE Arabidopsis plants, the induction of *PAD3, RetOx* and *PR1* was noticed, whereas induction of *PR1* was noticed in AnAXE plants (Figure 4A). These results indicate that ectopic expression of AnAXE and AnRAE might have caused different CW alterations, and each are able to trigger specific defense pathways.

A previous study on whole-genome transcript profiling and RT-qPCR analyses showed that *PAD3, RetOx* and *WR3* were rapidly and highly up-regulated upon exposure to OGs and other elicitors [80,81]. PAD3 was reported to be involved in the biosynthesis of an antimicrobial phytoalexin (camalexin) to defend against fungal pathogens [80,82]. WR3 was established to be involved in JA-independent wound signal transduction of plant defense [83]. Results showed that RetOx expression is induced mainly by DAMPs and results in the production of H_2_O_2_, which in turn might restrict/kill the pathogens locally and act as a signal for the induction of defense pathways distantly [84]. In agreement with the above finding, the obtained results here showed that increased accumulation of H_2_O_2_ observed in AnRAE, AnAXE/AnRAE, AnAXE/AnFAE and AnRAE/AnFAE plants positively correlates with induction of *RetOx* transcript level observed in RT-qPCR (Figure 4 and Figure 5).

Further, the DAB staining showed that AnRAE/AnFAE plants had higher H_2_O_2_ accumulation (Figure 5), and this could have produced higher resistance in AnRAE/AnFAE than any other plants (Figure 3). However, our pathogen infection assays showed that AnAXE/AnRAE plants (due to additive effect) were more resistant than AnRAE/AnFAE plants (due to compensatory effect), and resistance produced by AnRAE/AnFAE plants was found to be only equal to Col-0. The RT-qPCR results showed that the other two double-gene expression lines (AnAXE/AnRAE and AnAXE/AnFAE) also expressed nearly the same level of *RetOx* to that of the AnRAE/AnFAE plant. Apart from RetOx-mediated hydrogen peroxide expression, other pathway genes shown in our RT-qPCR data and many other unknown pathway genes might have combinedly contributed to pathogen resistance instead of depending only on hydrogen peroxide expression. The combined effect of many pathway genes might have overall contributed to the higher *B. cinerea* resistance seen in AnAXE/AnRAE plants than AnRAE/AnFAE, which is in agreement with the earlier finding that complexity of transcriptional programming and gene regulatory network are needed for Arabidopsis defense against *B. cinerea* [85]. This indicates that the involvement of CW-mediated defense pathway genes in protecting the plants against biotic and abiotic stress is highly complex and careful selection criteria is needed for gene stacking by considering all the aspects of molecular biology of plant–pathogen interactions.

Previously, it was shown that PDF1.2 was regulated by Jasmonic acid (JA), which is mainly involved in plant defense reactions against necrotrophic pathogens such as *B. cinerea* [86]. Our transcriptional analysis also revealed that the *PDF1.2* gene, encoding defensin protein in Arabidopsis, was highly induced in all the three double-transgenic overexpressor lines, whereas it was down regulated in the single transgenic lines (Figure 4). One possible explanation is that in the absence of infection, the deacetylation or deferuloylation caused by single-gene expression in transgenic plants seems to activate DAMPs and SA-related genes such as *PAD3, RetOx* and *PR1* at the expense of the Jasmonic acid-induced genes such as *PDF1.2* and *JR1*. In the double-gene expression lines, the combined effect of deacetylation and/or deferuloylation might have been perceived by the plants as a more serious loss of CW integrity to be managed with a more complete arsenal of weapons, including Jasmonic acid-related genes. Further in-depth research might be needed in the future to address the complex phenomenon of expression and involvement of genes in the CW-related defense pathway.

In our study, the *PR1* gene was found to be highly induced in transgenic AnAXE, AnRAE and AnAXE/AnRAE plants, but not in plants with AnFAE (Figure 4). Interestingly, it correlates well with the pathogen resistance observed in these transgenic plants (Figure 3). Earlier, it has been shown that PR-1 family proteins are the most highly produced proteins upon pathogen attack, and most of the PR-1 proteins are secreted into the extracellular/apoplast region. PR-1 proteins play a vital role in plant defense, such as broad-spectrum antimicrobial compound, act as receptors for recognizing pathogen effectors, and signal molecules for salicylic acid-mediated disease resistance [87].

## 5. Conclusions

In conclusion, the results obtained in this research demonstrated the potential impact of the post-synthetic modification of CW polysaccharides by introducing CW-degrading enzymes. The CW polysaccharide modifications can trigger complex responses to defend the plants against both the biotic and abiotic stresses. This study also confirms and provides new evidence that the additive effect of a combination of different polysaccharide modifications can constitutively prime plant defense pathways even before pathogen infection and offers enhanced plant protection. However, it should be emphasized that the involvement of CW-mediated defense is highly complex; therefore, selection of complimentary genes should be based on careful consideration of all aspects of molecular biology of plant–pathogen interactions. Apart from the genes we used in the present study, numerous other genes for CW polysaccharide modifying enzymes are available and could be considered in the future. Stacking several CW degrading enzymes with different specificity towards various CW components to induce diverse signal pathways could be a promising direction in basic research on molecular biology of CW integrity signaling, as well as useful in applied research as a tool to generate varieties of valuable crops with improved stress resistance and biomass quality.

## Data Availability

The data presented in this study are available upon request from the corresponding author.

## Supplementary Materials

The following are available online at www.mdpi.com/article/10.3390/biology10101070/s1, Figure S1: Gibson assembly scheme for the generation of recombinant vector, pCAMBIA-UBQ10-AnFAE-CFP used in this study. The expression cassette contains the UBQ10 promoter, *AnFAE-CFP* fusion gene, and the 35S terminator were ligated together to the backbone from the pCAMBIA-1300-MCS expression vector using one-step Gibson assembly. Similar strategy was used to clone *AnAXE* and *AnRAE* genes into pCAMBIA-1300-MCS, Figure S2: Real time-qPCR analysis of transcript level of transgenes in transgenic lines and WT plants (Col-0). RT-qPCR analysis was conducted to find out the transcript level of individual introduced transgenes in six dif-ferent transgenic lines (*AnAXE*, *AnRAE*, *AnFAE*, *AnAXE/AnRAE*, *AnAXE/AnFAE* and *AnRAE/AnFAE*). *ACTIN2* was used as a reference gene to normalize the data. The transcript data represents average and ± SD of three different independent transgenic lines for each construct. Asterisks indicate significant differ-ences between the mean transcript level among the transgenic plants and WT plants (Student’s t test, *p* < 0.05; *n* = 3), Table S1: List of primers used in this study (5’-3’).
