# Supplementary material for: Coexpression of Fungal Cell Wall-Modifying Enzymes Reveals Their Additive Impact on Arabidopsis Resistance to the Fungal Pathogen, Botrytis cinerea
Source: Biology (Basel). 2021 Oct 19;10(10):1070. doi: 10.3390/biology10101070 (PMC8533531; doi:10.3390/biology10101070)
Supplement: Supplementary file 1 [file biology-10-01070-s001.zip › biology-1410637-supplementary.pdf]

Supplementary Materials

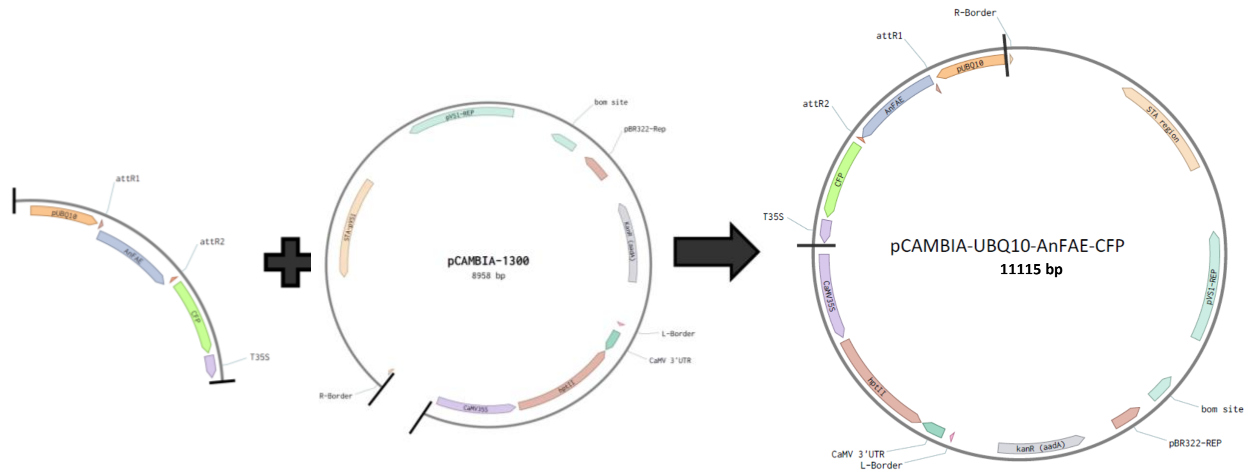

Figure S1. Gibson assembly scheme for the generation of recombinant vector, pCAMBIA-UBQ10-AnFAE-CFP used in this study. The expression cassette contains the UBQ10 promoter, *AnFAE-CFP* fusion gene, and the 35S terminator were ligated together to the backbone from the pCAMBIA-1300-MCS expression vector using one-step Gibson assembly. Similar strategy was used to clone *AnAXE* and *AnRAE* genes into pCAMBIA-1300-MCS.

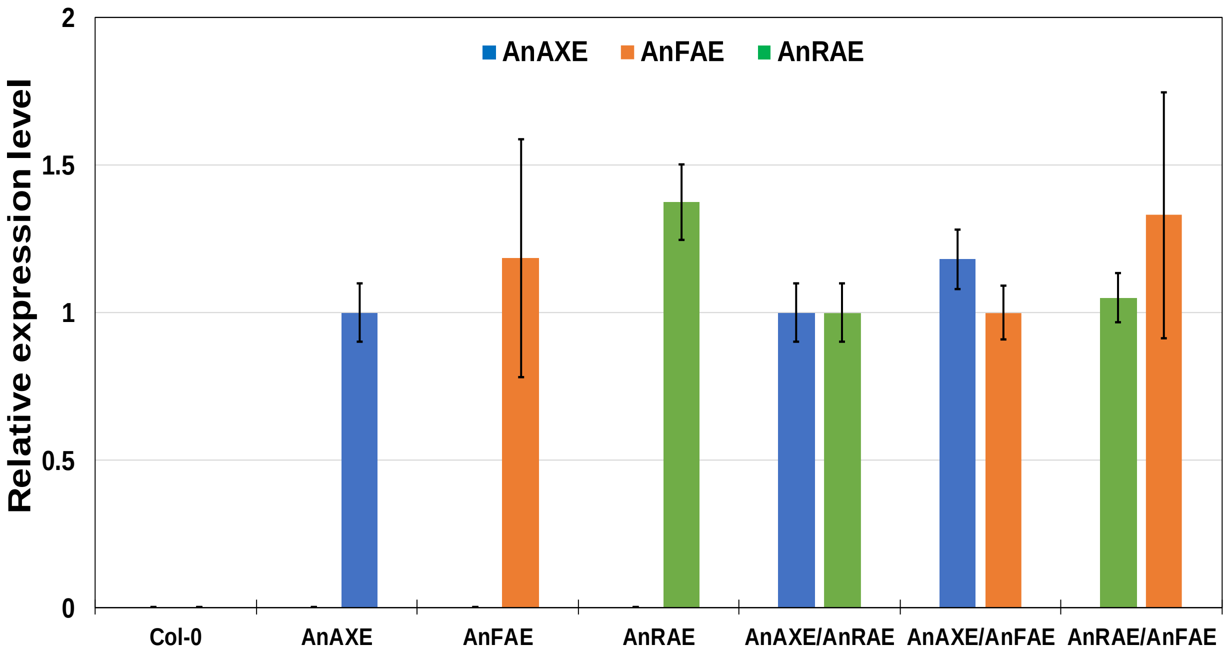

Figure S2. Real time-qPCR analysis of transcript level of transgenes in transgenic lines and WT plants (Col-0). RT-qPCR analysis was conducted to find out the transcript level of individual introduced transgenes in six different transgenic lines (*AnAXE*, *AnRAE*, *AnFAE*, *AnAXE*/*AnRAE*, *AnAXE*/*AnFAE* and *AnRAE*/*AnFAE*). *ACTIN2* was used as a reference gene to normalize the data. The transcript data represents average and ± SD of three different independent transgenic lines for each construct. Asterisks indicate significant differences between the mean transcript level among the transgenic plants and WT plants (Student's t test, $p < 0.05$; n=3).

Table S1. List of primers used in this study (5′-3′).

Name of the primer	Sequence of the primer
AtPR1-F	TCTAAGGGTTCACAACCAGG
AtPR1-R	CCTTCTCGCTAACCCACATG
AtPR5-F	GAGGATCGGGAGATTGCAAA
AtPR5-R	GTCAGGGCAAGCGTTCTTGA
AtbG2-F	GACGCAAATCTCGACTCGGT
AtbG2-R	TCTCTATAGCTTTCCCTGGC
AtPAD3-F	ACTCTGGGAAAACGCAGATG
AtPAD3-R	CTTTGGCTTCCTCCTGCTTC
AtJR1-F	GTGTCGGGCTACTATGACAA
AtJR1-R	GGGCGCAACATTGACTCCAA
AtWR3-F	TTCGTGCCTACGCGGTTGAT
AtWR3-R	CTATCTTGGCCTTCCTCTTC
AtPGIP-F	CAGCTCAAGAATCTCGAGTT
AtPGIP-R	TCGATCCGGTTAAAGTCGAT
AtWRKY-F	CTAGAGACAATCCATCTCCA
AtWRKY-R	TGCTGCAACGGGTGTTGAAG
AtCYP-F	CAGCTGCACCACTTCTTGTT
AtCYP-R	CACCAGGACACGTTCTTCGT
AtRetO-F	AATGATGGATCGGATTCCGT
AtRetO-R	ACCGCTTGGATTGCTTCCAA
AtJaPDF1.2-F	TTGCTTCCATCATCACCCTT
AtJaPDF1.2-R	CACTTGGCTTCTCGCACAAC
AtActin2_F	GAAACCCTCGTAGATTGGCA
AtActin2_R	CTCTCCCGCTATGTATGTCGC
AnAXE-F	CGATCCACTACTGCACTGGAAC
AnAXE-R	GTTAGAGTTGACTGCGAGCTGAC
AnRAE-F	CCATGGCGTCCAGTACTCCTG
AnRAE-R	GTCGCCTGCTTGAAGGACGTC

AnFAE-F	GATGGCTACGACCCAAGCAAG
AnFAE-R	CAGCCAGCATTCAGACCGTTG
